# T cell Ca^2+^ microdomains through the lens of computational modeling

**DOI:** 10.3389/fimmu.2023.1235737

**Published:** 2023-10-04

**Authors:** Diana C. Gil Montoya, Roberto Ornelas-Guevara, Björn-Philipp Diercks, Andreas H. Guse, Geneviève Dupont

**Affiliations:** ^1^The Calcium Signalling Group, Department of Biochemistry and Molecular Cell Biology, University Medical Center Hamburg-Eppendorf, Hamburg, Germany; ^2^Unit of Theoretical Chronobiology, Faculté des Sciences CP231, Université Libre de Bruxelles (ULB), Brussels, Belgium

**Keywords:** store-operated Ca^2+^ entry (SOCE), Orai, 3D computational model, Ca^2+^ microdomains, nicotinic acid adenine dinucleotide phosphate (NAADP)

## Abstract

Cellular Ca^2+^ signaling is highly organized in time and space. Locally restricted and short-lived regions of Ca^2+^ increase, called Ca^2+^ microdomains, constitute building blocks that are differentially arranged to create cellular Ca^2+^ signatures controlling physiological responses. Here, we focus on Ca^2+^ microdomains occurring in restricted cytosolic spaces between the plasma membrane and the endoplasmic reticulum, called endoplasmic reticulum-plasma membrane junctions. In T cells, these microdomains have been finely characterized. Enough quantitative data are thus available to develop detailed computational models of junctional Ca^2+^ dynamics. Simulations are able to predict the characteristics of Ca^2+^ increases at the level of single channels and in junctions of different spatial configurations, in response to various signaling molecules. Thanks to the synergy between experimental observations and computational modeling, a unified description of the molecular mechanisms that create Ca^2^^+^ microdomains in the first seconds of T cell stimulation is emerging.

## Ca^2+^ signaling research: from global to local

Ca^2+^ signaling is the major intracellular signaling system found in almost all cell types in the animal kingdom, but also extending to plant cells and fungi (1, 2). Ca^2+^ signaling requires Ca^2+^ gradients across organellar membranes and across the plasma membrane (PM). ATP-driven Ca^2+^ pumps, such as sarco-/endoplasmic reticular Ca^2+^ ATPase (SERCA) and plasma membrane Ca^2+^ ATPase (PMCA), build up such Ca^2+^ gradients allowing for rapid Ca^2+^ release from organellar Ca^2+^ stores and/or Ca^2+^ entry across the plasma membrane upon cell stimulation.

Global Ca^2+^ signaling in the form of an increase in the free cytosolic Ca^2+^ concentration ([Ca^2+^]_i_) throughout the cytosol, most often extending into the nucleus, was first described in the 1980s (3). Such experiments were first performed in cell suspensions analyzed in a fluorimeter; however, with the advent of microscopic imaging technology, global Ca^2+^ signaling was also observed in single cells (4). With the increasing spatio-temporal resolution of microscopes and cameras, analyses of the dynamics of local Ca^2+^ signaling has become possible. In particular, the advent of confocal microscopy and the development of super-resolution imaging methods were major steps to allow for very high resolution in both space and time. Further, novel deconvolution approaches allow to increase signal-to-noise ratio even in dim raw images, adding another important step to disentangle fast local Ca^2+^ signaling events (5).

Recently, we reviewed functions and mechanisms of formation of Ca^2+^ microdomains (6). In summary, Ca^2+^ microdomains in which the rise in concentration reaches a few hundred nanomolar and extending on 1-3 microns were found in different cell types. Pioneering work by Peter Lipp, Martin Bootman and colleagues revealed elementary Ca^2+^ signal events, likely representing Ca^2+^ release from clusters of either ryanodine receptors (RYR), termed ‘sparks’ or d-*myo*-inositol 1,4,5-trisphosphate receptors (IP_3_R), termed ‘puffs’. Smaller fundamental Ca^2+^ signal events that relate to Ca^2+^ release from single RYR, termed ‘quarks’ or IP_3_R, termed ‘blips’ were also described (7–10). In addition to these local Ca^2+^ release events, Ca^2+^ sparklets were defined as local signals resulting from brief openings of plasma membrane Ca^2+^ channels (11–13).

More recently, Ca^2+^ microdomains occurring in confined spaces between closely apposed membranes have been described. Locally elevated Ca^2+^ concentrations arise around mitochondria-associated ER membranes (MAM) that provide privileged platforms for Ca^2+^ transfer from the ER into mitochondria (14). Similarly, sharp and short-lived Ca^2+^ increases can sometimes be observed at the ER-PM contact sites or junctions. These signals are dependent on Ca^2+^ entry from the extracellular medium through store-operated Ca^2+^ entry (SOCE). The latter mechanism relies on the stromal interaction molecules (STIM1 and/or STIM2) that are ER Ca^2+^ sensors regulating the activity of the Orai1 PM Ca^2+^ channels. Thus, upon local depletion of the ER Ca^2+^ store, Ca^2+^ dissociates from STIM, which allows recruitment of Orai1 to the junction and their gating (15–18). These channels are also referred to as “Ca^2+^ release activated channels (CRAC)”. The ER-PM junctions are areas in the cytosol where the ER gets really close to the PM, with a depth of approximately 15 nm and an extension of ~200 nm (18).

As proposed by 19, Ca^2+^ microdomains can be defined as “*subcellular, rapid and localized high Ca^2+^ concentration regions that develop near open Ca^2+^ channels at the plasma membrane or internal stores”*. Key factors that determine their specificity in triggering different signaling pathways in different cell types are their *amplitude, frequency* and *duration*. Variability in these factors leads to specific forms of global Ca^2+^ increases. The detailed characterization of Ca^2+^ microdomains is limited by the spatio-temporal resolution of available imaging techniques. Computational modelling has therefore much been used to further investigate their molecular origin. These studies rely on the theoretical tools that have been developed to describe global Ca^2+^ signaling (20), although they face specific problems related to the confined geometry and the steep gradients that need to be simulated (18). Moreover, stochastic approaches are sometimes required to take into account the local fluctuations in concentrations due to the small numbers of Ca^2+^ channels present in the microdomains (21–25).

Local Ca^2+^ entry via SOCE activates different cellular responses in different cell types depending on their spatio-temporal profiles. These include transcription factor stimulation in B cells by high amplitude Ca^2+^ spikes, NFAT activation in T cells by a sustained low amplitude signal and exocytosis stimulation in non-excitable cells by spontaneous repetitive short Ca^2+^ signals. It is known that the modulation of such localized Ca^2+^ signals involves clusters of ER (IP_3_R or RYR) or PM (Orai1) Ca^2+^ channels, but the detailed interplay between these channels, including their molecular effectors, remain to be fully characterized.

## Ca^2+^ microdomains in T cells

Although most of the Ca^2+^ toolbox molecules, e.g. second messenger forming and degrading enzymes, Ca^2+^ mobilizing second messengers and their receptors, Ca^2+^ channels, Ca^2+^ storing organelles, and Ca^2+^ pumps are often ubiquitously expressed, the specific configuration of a particular cell type determines the exact mechanism of both local and global Ca^2+^ signaling. Thus, in this review, we will concentrate on a particular cell type that has been extensively characterized regarding mechanisms involved in the formation of Ca^2+^ microdomains, mammalian T-lymphocytes. The main molecules involved are listed in **Table 1**, for ease of reading. Over the past 10 years, Ca^2+^ microdomains at the ER-PM junctions have been extensively studied in these cells. Despite disadvantages of this cell model, e.g. small diameter of about 6-7 μm and a large nucleus as compared to the cytosol, as well as spherical shape with many stacked z-layers that potentially result in image blurring, development of a high-resolution Ca^2+^ imaging method combined with image refining by off-line deconvolution resulted in spatio-temporal resolution of 368 nm and 25 ms (26). Further, activation of the T cell receptor (TCR)/CD3 complex and co-stimulation of CD28 by small beads coated with monoclonal antibodies against CD3 and CD28 allowed for point-shaped activation of few TCR/CD3 complexes, thereby mimicking activation during formation of an immune synapse (26).

**Table 1:** Main actors involved in T cells Ca^2+^ microdomains.

Acronym	Complete name	Importance for Ca^2+^ microdomains in T cells
DUOX2	dual NADPH oxidase 2	enzyme that catalyzes NAADP formation using NAADPH as substrate
FAK	focal adhesion kinase	a key mediator of integrin signaling. When phosphorylated, it activates PLCγ
IP_3_	d-*myo*-inositol 1,4,5-trisphosphate	second messenger that binds to IP_3_R to release Ca^2+^ from ER
IP_3_R	inositol trisphosphate receptor	Ca^2+^ channel located in the ER membrane, which releases Ca^2+^ into the cytosol upon binding of the second messenger IP_3_
NAADP	nicotinic acid adenine dinucleotide phosphate	second messenger triggering Ca^2+^ release through RYR1 located in the ER membrane
Orai		a class of membrane Ca^2+^ channels interacting with STIM proteins, which are themselves activated by depletion of Ca^2+^ in the ER. Orai channels are a principal component of store operated Ca^2+^ entry (SOCE).
PLC	phospholipase C	enzyme that catalyzes the synthesis of IP_3_ from phosphatidylinositol 4,5-bisphosphate
PMCA	plasma membrane Ca^2+^ ATPase	transports Ca^2+^ out of the cell actively; major Ca^2+^ extrusion pathway in T cells
RYR	ryanodine receptor 1	Ca^2+^ channel located in the ER membrane, which opens and releases Ca^2+^ to the cytosol upon NAADP binding.
SERCA	sarco/endoplasmic reticulum calcium ATPase	transports Ca^2+^ into the ER actively
STIM	stromal interaction molecule	protein spanning the ER membranes with domains sensing luminal Ca^2+^ concentration and for activation of Orai channels
TCR/CD3	T cell receptor/cluster of differentiation 3	complex formed by T cell Receptor (TCR) and cluster of differentiation 3 (CD3) proteins, which activates downstream signals, amongst which NAADP is formed

**Figure 1:**
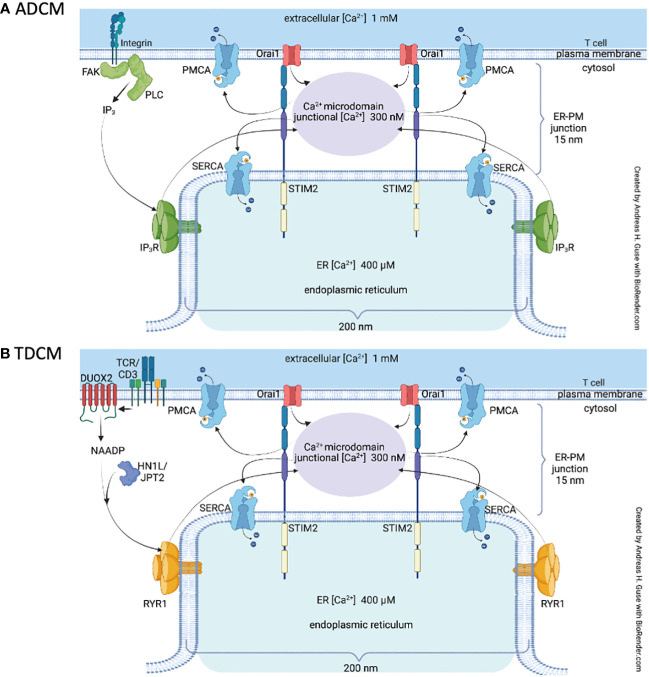
Local Ca^2+^ signaling in T cells Schematic representation of the main features involved in T cell adhesion-dependent Ca^2+^ microdomains, ADCM **(A)** and TCR/CD3/CD28-dependent Ca^2+^ microdomains, TDCM **(B)**. For abbreviations see text and **Table 1**.

So far, two different signaling mechanisms involved in formation of Ca^2+^ microdomains in T cells were identified: (i) adhesion-dependent Ca^2+^ microdomains (ADCM) evoked by integrin-mediated IP_3_ signaling and subsequent SOCE (**Figure 1A**), or (ii) Ca^2+^ microdomains evoked by TCR/CD3/CD28 stimulation (TDCM), proceeding via nicotinic acid adenine dinucleotide phosphate (NAADP) formation by dual NADPH oxidase 2 (DUOX2), and activation of RYR1 via the NAADP receptor hematological and neurological expressed 1-like protein (HN1L)/jupiter microtubule associated homolog 2 (JPT2). Hereafter, ADCM and TDCM will refer to the spatially restricted and short-lived Ca^2+^ increases occurring in the so-called microdomains. TDMC are amplified by SOCE (27) (**Figure 1B**) and purinergic signaling via P2X4 and P2X7 (28).

Long before the observation of ADCM, Weissmann and colleagues described global Ca^2+^ responses in Jurkat T cells that were triggered by integrin-mediated adhesion to extracellular matrix (ECM) proteins (29). These Ca^2+^ signals were dependent on the phosphorylation of focal adhesion kinase (FAK). FAK is thought to be a key component of the integrin-mediated signaling cascade (30) and evokes Ca^2+^ signals selectively at membrane rafts during the cell–matrix adhesion process (31). Stimulation of FAK subsequently activates phospholipase C-γ (PLC-γ) (32), which in turn generates IP_3_ leading to a Ca^2+^ release from the ER by targeting IP_3_ receptors (33). Local decrease in ER Ca^2+^ concentration then activates SOCE. Indeed, locally restricted Ca^2+^ microdomains in the absence of TCR stimulation were shown to be dependent on the expression of Orai1 and STIM1/2 (27). Interestingly, the NAADP/RYR1 axis was not involved in this process in primary T cells (27). However, another Ca^2+^ mobilizing second messenger, termed cyclic ADP-ribose (cADPR), which evokes Ca^2+^ signals through ryanodine receptor type 3 (34), was involved in global Ca^2+^ responses mediated by integrins in Jurkat T cells (35). Purinergic signaling via P2X4, is also involved in the formation of TCR independent Ca^2+^ microdomains in primary T cells (28). However, whether integrin mediated Ca^2+^ signaling evokes ATP release followed by purinergic signaling via P2X4 in an autocrine manner is not yet clear. Concerning the physiological role of adhesion-dependent Ca^2+^ microdomains, it has been suggested that they may facilitate full TCR-mediated activation (36) given that T cells form adhesive interactions with endothelial cells or ECM proteins during their migration from blood vessels into inflamed tissues (37).

TDCM require functional NAADP signaling in T cells (**Figure 1B**). A crucial role of NAADP in T cells was discovered by showing global and local Ca^2+^ signaling upon NAADP microinjection (38, 39; Dammermann & Guse; 2005; 26, 40). RYR was identified as a major Ca^2+^ channel in NAADP signaling in T cells (39; 41; 26, 27, 40). In addition, NAADP evoked Ca^2+^ entry secondary to Ca^2+^ release was also shown for the first time in T cells (39). The mechanism of NAADP evoked release of Ca^2+^ from intracellular Ca^2+^ stores started to being disentangled by the discovery of small cytosolic NAADP receptor/binding proteins, firstly as 22/23 kDa band labeled by a NAADP-photoaffinity probe (42) and finally by identifying the NAADP receptor/binding protein as HN1L/JPT2 in both T cells (43) and erythrocytes (44). Of note, Lsm12 was identified as another NAADP binding protein (45). HN1L/JPT2 co-localized with RYR in T cells as shown by STED super-resolution microscopy and co-immunoprecipitation (43), suggesting that NAADP bound to HN1L/JPT2 directly activates RYR, as hypothesized in the unifying hypothesis for NAADP action (46). The latter also includes, as further potential link between NAADP and activation of ion channels, the possibility of activation of two-pore channels (46). In addition to the Ca^2+^ releasing mechanism of NAADP, formation and degradation of NAADP are rather important for this signaling pathway. Along these lines, it was reported for T cells that NAADP formation within seconds upon TCR/CD3 stimulation (47) proceeds by oxidation of NAADPH to NAADP, catalyzed by DUOX2 (48). Rapid reduction of NAADP to NAADPH may be catalyzed in intact cells by glucose 6-phosphate dehydrogenase, that, together with DUOX2, constitutes a redox cycle for fast formation of NAADP and its ‘storage’ as inactive reduced NAADPH (48). Full degradation of NAADP is catalyzed by CD38 (to 2-phospho-ADPR) or by alkaline phosphatase (to NAAD) (49, 50). Crucial importance of NAADP signaling for Ca^2+^ microdomain formation in T cells was demonstrated by gene deletion or silencing of *Duox2, Hn1l/Jpt2*, or *Ryr1* (26, 27, 43, 48).

Gene deletion of the major proteins of SOCE in T cells, STIM1 and 2, and Orai1, revealed that in addition to NAADP evoked release of Ca^2+^, also SOCE is essentially required for the formation of the local Ca^2+^ microdomains (27). Very recently, another amplification system for Ca^2+^ increases inside microdomains in T cells was discovered as autocrine ATP evoked activation of purinergic Ca^2+^ channels P2X4 and P2X7 (28).

TDCM are observed as soon as a few hundreds of millisecond post TCR/CD3/CD28 stimulation and last for approx. 15 to 25s (**Figure 1B**). Among the other initial signaling pathway active in T cells in this very early period of time, tyrosine phosphorylation of target proteins is certainly outstanding, e.g. by the tyrosine kinases ZAP70 and/or p56^lck^ (51). Experimental evidence for their involvement in TDCM was obtained: NAADP formation evoked by TCR/CD3 stimulation was completely blocked by tyrosine kinase inhibitor genestein (47). In contrast, during the first 15s, levels of IP_3_ and diacylglycerol, both products formed from phosphatidylinositol 4,5-bisphosphate by phospholipase C-γ (52) are still quite low (53, 54) and do not significantly impact on Ca^2+^ microdomains, as demonstrated, at least for IP_3_ evoked Ca^2+^ release, by IP_3_ antagonism (26). Later on, between approx. 20 and 30s post TCR/CD3/CD28 stimulation, TDCM merge into global Ca^2+^ signaling. Major mechanisms involved in global Ca^2+^ signaling are (i) IP_3_ evoked Ca^2+^ release between approx. 30s and 10 to 15 min (53), (ii) cyclic ADP-ribose mediated Ca^2+^ release started between 5 and 10 min and lasting for at least 60 min (55). SOCE via STIM1 and Orai1 is the major Ca^2+^ entry mechanism secondary to ER Ca^2+^ store depletion by IP_3_ and cyclic ADP-ribose [reviewed in (56).

## ER-PM Ca^2+^ microdomains in T cells: modeling assumptions

As detailed above, current techniques in microscopy allow to analyze both global Ca^2+^ dynamics and localized Ca^2+^ events. These observations however raise numerous questions about the molecular mechanisms involved in the foundation of Ca^2+^ microdomains. Some of these questions are best addressed by computational modelling, which is made possible thanks to an impressive quantitative characterization of the properties of ER-PM Ca^2+^ microdomains. In particular, models allow to predict the number and spatial arrangement of the channels inside the junction, which remains inaccessible with current experimental techniques.

A few studies have focused on modelling Ca^2+^ microdomains due to SOCE. In a series of pioneering studies (57–59), Ca^2+^ changes at the immunological synapse have been modelled in 1D and later in 3D. These early models focused on the impact of mitochondria on the Ca^2+^ influx through Orai, but most of them did not specifically address the detailed conditions related to the small-scale microdomains in the ER-PM junctions. To assess the impact of mitochondria relocalization to the immunological synapse on the spatial distribution of Ca^2+^ inside the cell, (60) developed a whole cell model considering specific geometries for mitochondria. The model predicted that the experimentally observed locations of this Ca^2+^-exchanging organelle, moving close to the PM, can account for the increase in Ca^2+^ around a 100 nm radius cluster of ORAI channels forming a Ca^2+^ microdomain. In the simulations, such local Ca^2+^ increases occur in conditions of massive ER Ca^2+^ depletion that correspond to situations encountered a few minutes after T cell stimulation, when mitochondria start to move along the cytoskeleton. In this study, the channel locations in the PM, ER or mitochondrial membranes are not specified and Ca^2+^ fluxes are homogeneously distributed across these membranes. The detailed impact of Ca^2+^ channel locations on the spatio-temporal profiles of Ca^2+^ changes inside the ER-PM junctions were analyzed in 1D by (18) and modelled in 3D by (61, 62) and (63, 64). In this section, we describe these models and their main relying assumptions, while their physiological outcomes are presented in the next section.

Model building can be divided in three steps. First, the biologically relevant variables that the model aims at simulating must be selected. Second, the spatial geometry in which these variables are evolving have to be defined, including the locations of organelles, channels, pumps, transporters, etc. Finally, well-chosen rate equations establish how the selected variables evolve in time in the elected sub-cellular configuration. As compared to global cell model, simulations of microdomains call for specificities especially when located around the mouth of a channel (20).

Concerning the choice of the variables, the formation of Ca^2+^ microdomains involve Ca^2+^ movements in the junctions, in the ER portion just beneath the junction (triggering SOCE) and in the extracellular medium. Given that the latter can be seen as an infinite source of Ca^2+^, its concentration can be considered as constant. Thus, Ca^2+^ concentrations in the junction and in the sub-PM ER compartment represent key variables. Because they dynamically regulate the junctional Ca^2+^ concentration, buffers should in principle be taken into account. Most models however ignore the effect of buffers because of the short time and length scales involved in microdomain formation. Because of the high Ca^2+^ concentrations and the small volume of the junction, local Ca^2+^ buffers are expected to be saturated (62, 65, 66). Similarly, Ca^2+^ diffusion is assumed to occur in an unbuffered cytosol (*D_c_* = 220 μm^2^s^-1^). When considering the relation between cell adhesion/stimulation and Ca^2+^ signaling, IP_3_ and NAADP should also be involved because they modify ER and junctional Ca^2+^ by affecting the opening states of the IP_3_Rs and RYRs, respectively. Although NAADP mobilizes lysosomal Ca^2+^ (67), the involvement of this organelle in the formation of T cells Ca^2+^ microdomains remains to be established (68). Instead of considering [IP_3_] and [NAADP] explicitly, their effects can be modelled by changing the opening states of their receptors directly (63, 64). This simplification is however not valid when considering the transition from local to global Ca^2+^ signaling which involves considering diffusion and metabolism of the messengers. These variables and their interplays are schematically shown in **Figure 1**.

**Figure 2:**
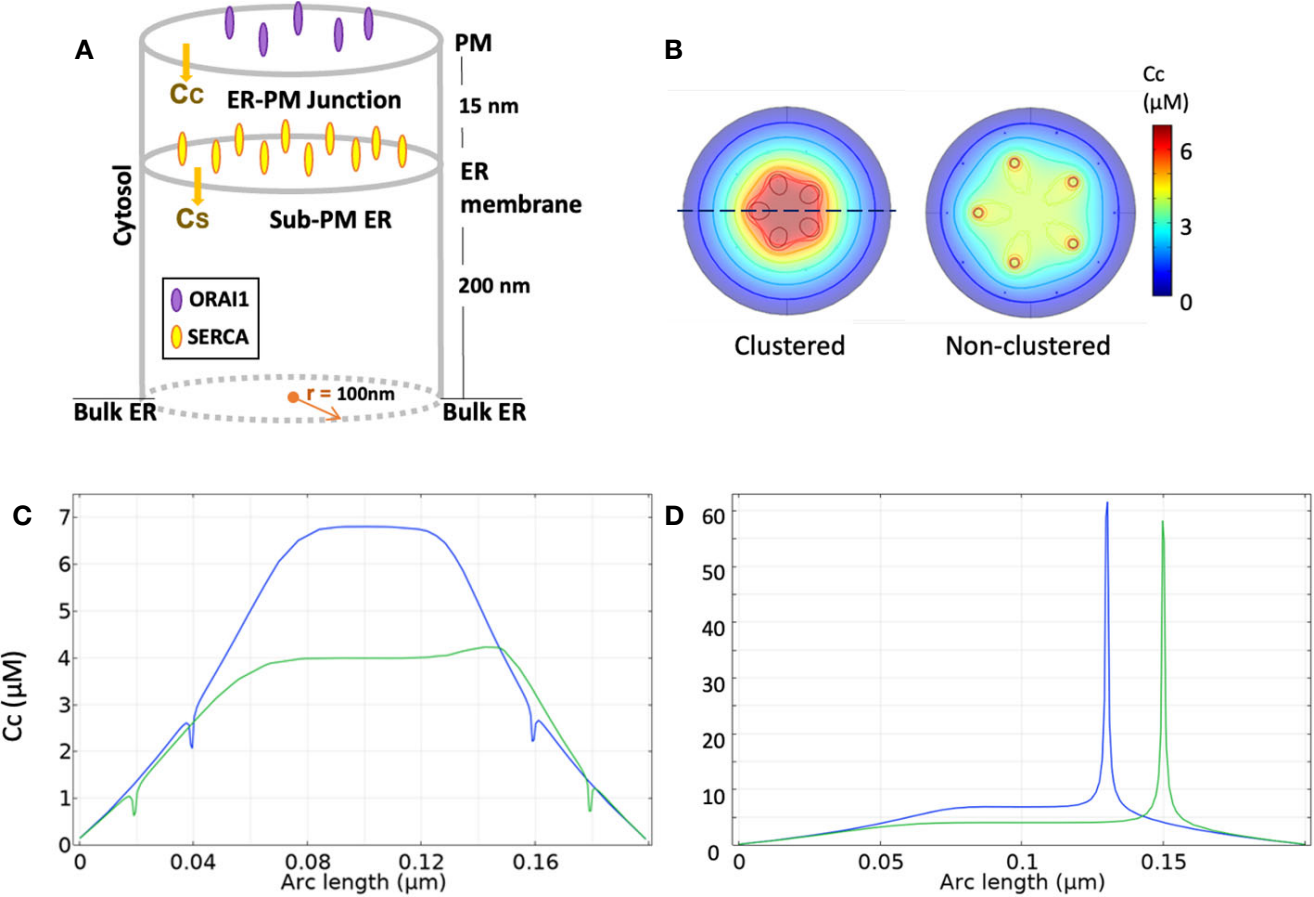
Simulation of Ca^2+^ signaling at ER-PM junctions, according to McIvor et al. (62) **(A)** Three-dimensional schematic view of the model geometry. **(B)** Ca^2+^ profiles at the ER membrane after 1 ms opening of 5 Orai channels under an Orai clustered (left) configuration with an inter-channel distance of 39 nm and a non-clustered (right) one with a 65 nm distance. **(C)** Ca^2+^ spatial signature along the dotted line in B for the clustered (blue line) and non-clustered (green line) configuration. Small decreases in junctional Ca^2+^ concentration can be seen near SERCAs. **(D)** Ca^2+^ spatial signature along the dotted line at the PM for the clustered and non-clustered configuration. All numerical simulations, initially run by McIvor et al. (62) using Green’s functions, are reproduced with COMSOL Multiphysics.

To predict the exact spatio-temporal dynamics underlying Ca^2+^ increases in microdomains, Ca^2+^ fluxes and diffusion must be simulated in a realistic computational description of the ER-PM junction configuration. A typical configuration, used with slight variations in (61, 62) and 63); 64), is schematized in **Figure 2A**. ER-PM junctions are regions of the cell where both membranes are closely apposed and correspond to the locations where STIM and Orai co-localize upon store depletion. In resting T cells, STIM2 pre-formed complexes with Orai localize at the ER-PM junctions (27). The distance between the two membranes has been estimated to be 10-20 nm in depth and ~200 nm in length (15, 18, 69, 70). This flat cylinder defines the junctional space, surrounded by the cytoplasmic space where Ca^2+^ concentration is assumed to remain constant given the limited amount of Ca^2+^ ions involved in the formation of microdomain. Similarly, the luminal space is arbitrarily divided in two regions: one close to the membrane facing the ER in which [Ca^2+^] changes dictate SOCE dynamics and one further away where [Ca^2+^] is assumed to remain constant due to fast replenishment from the rest of the ER.

**Figure 3:**
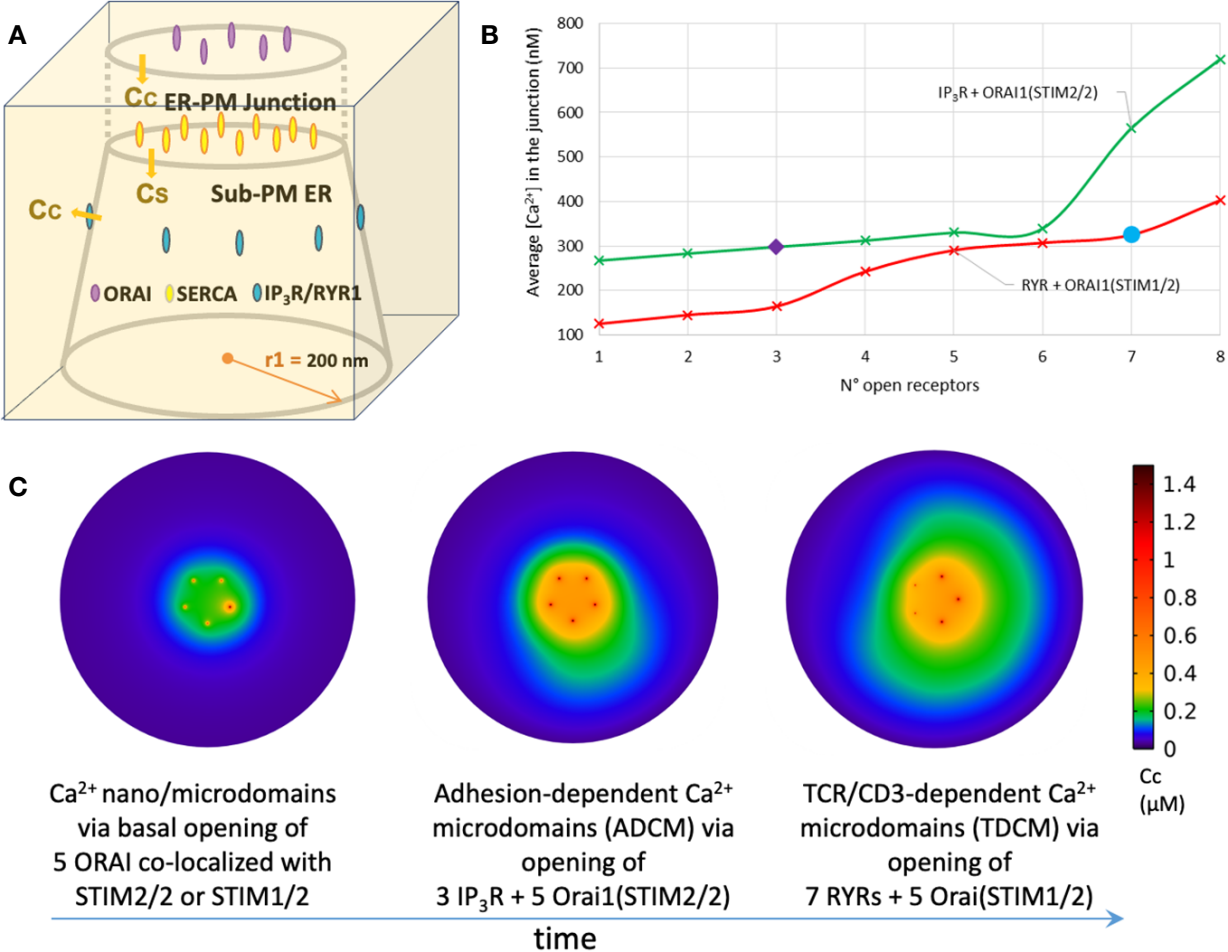
Simulation of T cell Ca^2+^ signaling at single ER-PM junctions **(A)** Three-dimensional schematic view of the model geometry that simulates the interplay of Orai1 and IP_3_R during adhesion-dependent Ca^2+^ microdomains (ADCM) in Gil et al. (63). **(B)** Evolution of the average Ca^2+^ concentration reached in the ER-PM junction with the number of open IP_3_Rs (green line) or RYRs (red line). SOCE relies on STIM2/2 homotetramers when local depletion in ER Ca^2+^ results from IP_3_Rs opening and on STIM1/2 heterotetramers when local depletion in ER Ca^2+^ results from RYRs opening. Three IP_3_Rs are needed to reproduce experimental observed adhesion-dependent Ca^2+^ microdomains (purple diamond) and seven RYRs are needed to reproduce experimental observed TCR/CD3-dependent Ca^2+^ microdomains (blue circle) showed in Diercks et al. (27). Simulations are performed as in Gil et al. (63, 64) and (2022) **(C)** Ca^2+^ profiles at the PM in the proposed T cell transition mechanism between quiescent adherent cells (t < 0, unstimulated T cell) to early activation (0 < t < 15s, first 15 seconds after stimulation). See text for detail. Simulations are performed as in Gil et al. (63, 64).

Five Orai channels are placed in the PM, as estimated for Jurkat T cells (18). They are arranged on a ring with an inter-channel distance of 47 nm (61). It should be noticed that the number of Orai channels in the junction is cell-dependent, as one channel per junction is reported for HeLa cells (71). The number of SERCA pumps present in the junction remains unknown and was arbitrarily set to 10 in in (61, 62 and (63, 64). In the whole cell model of (60), 10^5^ SERCA’s spread over the entire surface area of the ER, which would also correspond to ~10 pumps per ER-PM junction assuming a homogenous distribution. However, model outcomes are practically independent of the numbers of SERCA’s that are found to exert little influence on the spatio-temporal dynamics of microdomain formation. In line with this finding, SERCAs were not included in the first studies of Ca^2+^ changes at the immunological synapse (57–59). IP_3_Rs and RYRs with a well-defined spatial distribution were explicitly considered by Gil et al. (63, 64), because of the focus of these studies on SOCE possibly induced by moderate, local increases of IP_3_ and NAADP. The spatial arrangement of the IP_3_Rs (see **Figure 3A**) mimics the observations of (72) in HeLa cells, where clusters of IP_3_Rs reside alongside the junction and facing the PM. In the absence of specific information, the same arrangement was also considered for RYRs. Indeed, when RYRs are placed inside the junction, which would correspond to the situation encountered in the cardiac dyadic clefts (73), simulated Ca^2+^ microdomains do not correspond to observations [see next section, (64).

How Ca^2+^ ions evolve in space and time in the ER-PM junction is fixed by partial differential equations (PDE), or by a stochastic reaction-diffusion scheme such as, for example, the Gillespie’s algorithm (74). Given a basal [Ca^2+^] in the 10-100 nM range and a volume of ~10^-17^ - 10^-15^ liter, the number of Ca^2+^ ions in the ER-PM junction is between 0.06 and 60. Because the magnitude of the concentration fluctuations is inversely proportional to the number of ions, it is expected that stochastic fluctuations arising from molecular interactions and diffusion do not average out, thus calling for the requirement of stochastic simulations. All the studies mentioned here above however used PDE’s. This huge simplification is based on previous studies that have compared stochastic and deterministic modelling of Ca^2+^ dynamics in other confined cellular subspaces, such as a dendritic spine or a cardiac dyadic cleft. These works have indeed shown that for an appropriate range of physiological relevant parameters, stochastic Ca^2+^ signaling is well described using a deterministic model (75). This conclusion holds as long as the gating of the channels is not influenced by the ions that they transport, as it would be the case in the presence of Ca^2+^-induced Ca^2+^ release (76–78). Fast and mobile buffers also increase the magnitude of the fluctuations in the microdomain (79). When modeling Ca^2+^ microdomains occurring at the ER-PM junction on time periods of a few seconds following T cell stimulation, these conditions are met, and a deterministic description is appropriate. The resolution of the PDE’s remains challenging for several reasons, including the steep concentration gradients or the existence of widely different time scales. The numerous computational studies of Ca^2+^ “puffs” and “sparks” (21–24, 80, 81) have also paved the way for the modelling of ER-PM Ca^2+^ microdomains. However, for puffs and sparks, there is no barrier for the diffusion of Ca^2+^ flowing through the ER Ca^2+^ channels. In contrast, Ca^2+^ microdomains in dyadic clefts share with ER-PM junctions the characteristic of being embedded between two closely apposed membranes and many conceptual problems related to the deterministic Ca^2+^ dynamics in such restricted spaces have been formally addressed in this case (75, 82). It is also possible to resort to compartmental model. Wieder et al. (78) have summarized the evolution of numerical simulation techniques developed until recently, which accurately approach the different spatial and temporal scales found in the analysis of Ca^2+^ microdomain formation. For T cell Ca^2+^ microdomains, McIvor et al. (62) resorted to Green functions while Gil et al. (63, 64) took profit of the high accuracy of the COMSOL Multiphysics software (83) [COMSOL Multiphysics^®^]. Thanks to its powerful mesh dissection, solution methods and abundant post process operation, this software offers a trustable and relatively easily tractable way of modelling multiscale cellular processes.

## Modelling of the formation of T cell Ca^2+^ microdomains: interplay between Orai1, IP_3_R and RYR1

Together with the increasingly accurate observations of Ca^2+^ dynamics in the ER-PM junctions, modelling studies have allowed to dissect Ca^2+^ signaling in these localized domains, and in particular the nature, number and spatial arrangement of the Ca^2+^ transporting channels that are at play at various stages following T cell stimulation. Samanta et al. [2015] were the first to provide a quantitative analysis of the Ca^2+^ profiles upon SOCE activation in a realistic 3D ER-PM junction. They showed that Orai clustering enables the Ca^2+^ sensors located on the ER surface to be exposed to Ca^2+^ levels considerably higher in the junction than in the nearby cytoplasm. In the same line, (62) focused on the Ca^2+^ spatial signatures in the ER-PM junction created by two different spatial arrangements of Orai channels and their impact in the ER refilling process: firstly, with an inter-channel distance of 39 nm (clustered) and secondly, of 65 nm (non-clustered), as shown in **Figure 2B**. They concluded that the spatial arrangement of the Orai channels significantly impacts Ca^2+^ changes in the junction. When clustered, junctional Ca^2+^ concentration rises from an initial value of 100 nM to 7 μM in the center of the junction, while this increase is limited to 4 μM in the non-clustered configuration (**Figure 2C**). In contrast, these changes do not impact on the rates of ER refilling because of the low K_D_ of SERCA pumps (~300 nM). Orai channels are assumed to remain open with a constant current of 2.1 fA (18) across each channel. At the mouth of the Orai channel Ca^2+^ concentrations around 60 μM are predicted (**Figure 2D**). The Ca^2+^ junctional concentration reaches its steady state already after 0.25ms, which highlights the temporal reactivity created by this spatial arrangement of channels and membranes.

Ca^2+^ microdomains in T cells appear under conditions of a full ER opposite to the conditions explored by (62). Yet they appear to be opposed to the PM suggesting that they originate directly at ER-PM junctions. Thus, their computational investigation has been performed in the same geometry as that proposed in (62) and schematized in **Figure 2A**. Diercks et al. (27) quantitatively characterized the two different types of Ca^2+^ increases in microdomains that precede global Ca^2+^ signaling in T cells. The first ones, termed ADCM, evoked by an interplay between IP_3_R and/or SOCE and triggered by adhesion, are characterized by a duration of 44 ± 4 ms and a junctional average amplitude of 290 ± 12 nM. The second ones, termed TDCM, relying on RYR and SOCE and triggered by early TCR/CD3 stimulation, are much more frequent and have a duration of 64 ± 3 ms and a junctional average amplitude of 325 ± 11 nM [see 6, for review].

### Simulation of the formation of adhesion-dependent Ca^2+^ microdomains

In weakly adhesive, but otherwise unstimulated T cells, STIM2 largely co-localizes with Orai1, which suggests that STIM2 might be pre-clustered and ready to activate Orai1. Considering the high sensitivity of this STIM isoform to small changes in luminal Ca^2+^ concentration (84), it is highly plausible that SOCE contributes to the generation of ADCM. Still, our simulations predicted that Orai1/STIM2 activation in conditions of full ER cannot account on its own for the observed increase in junctional Ca^2+^ (63) indicating that probably an ER Ca^2+^ channel is also involved. Given that RYR1s are not involved in ADCM (27), an interplay between ORAI1 and IP_3_R, as suggested in (72), was proposed. This was found to be in agreement with the observation that adhesion triggers the phosphorylation of FAK and hence PLC activation and IP_3_ synthesis (27). To computationally evaluate the possible involvement of IP_3_Rs in the onset of ADCM, the eight IP_3_Rs located around the junction are opened gradually one at a time in a deterministic way at given time points (**Figure 3B**, green curve). Depending on the luminal Ca^2+^ concentration around its closest IP_3_R, each Orai1 opens in one of its four conductance states. This dependency of the open state on ER Ca^2+^ concentration follows a Hill function determined by STIM2 Ca^2+^ affinity. In order to reproduce ADCM observed experimentally (27), three open IP_3_Rs are needed together with a cluster of five Orai1 channels bound to STIM2 (**Figure 3B**, purple diamond). The cluster of Orai1 channels or of IP_3_R alone cannot account for the observed magnitude of the Ca^2+^ rise in the microdomain. Another interesting prediction of the model relates to the effect of changing the Ca^2+^ diffusion coefficient inside the ER. By default, a low value for this coefficient (*D_ER_* = 10 μm^2^s^-1^) was considered, to account for molecular crowding in the lumen and for the tortuosity of the tubular ER network (81). When *D_ER_* = 100 μm^2^s^-1^ is taken in the simulations, the amplitude of the Ca^2+^ increase and the spatial extent of the microdomains are much increased: opening of one IP_3_R already leads to a Ca^2+^ increase in the microdomain that exceeds experimental observations. Because of the faster replenishment at the channel mouth, the flux through the IP_3_R is indeed larger, which has a significant effect on junctional Ca^2+^ concentration. In other words, with the low value for *D_ER_*, Ca^2+^ release via IP_3_R is limited by local replenishment. SOCE activation does not compensate for release via the IP_3_R because of the ~30 times larger current of the latter.

### Simulation of the formation of TCR-dependent Ca^2+^ microdomains (TDCM)

In a similar way, Gil et al. (64) investigated how the interplay between Orai1 and RYR1 accounts for the formation of Ca^2+^ microdomains observed during the first 15 seconds after TCR/CD3 stimulation, termed TCR-dependent Ca^2+^ microdomains (TDCM). Following stimulation, NAADP signaling activates RYR1 and releases Ca^2+^ from the ER to the cytosol (40). Very recently, we identified hematological and neurological expressed 1-like protein (HN1L)/jupiter microtubule associated homolog 2 (JPT2) as NAADP receptor/binding protein that mediates activation of RYR1 upon NAADP binding (43); however, this step has not yet been included into the mathematical simulations. In parallel to increases of endogenous NAADP in the first seconds of TCR/CD3 stimulation, a higher co-localization of STIM1 with ORAI1 is observed in comparison to ADCM during which STIM2 co-localization predominates (27). This suggests that shortly after stimulation the main trigger of SOCE is Orai1 binding to STIM1 or most probably to heterotetramers of STIM1/2. Gil et al. (64) considered eight RYR1s around the junction, in the same way as described previously for IP_3_Rs. Indeed, if considered inside the junction as in cardiac myocytes (73), one receptor would already saturate the signal, contrary to experimental observations. To consider the effect of STIM1/2 binding, the dependency of Orai1 opening to local luminal Ca^2+^ concentration at the mouth of RYR1 is modified, with a lower Ca^2+^ sensitivity to depletion than that of STIM2 but higher than that of STIM1. In order to reproduce the observed TDCM, the opening of seven RYR1s together with a cluster of five Orai1 channels bounded to STIM1/2 is needed (**Figure 3B**, blue circle). Moreover, simulations foresee that Orai1 bound to STIM2 homotetramers or to STIM1/2 heterotetramers do not activate further than 21% of their full conductance under early local depletion conditions of the sub-PM ER, either across IP_3_R or RYR1. Thus, despite the involvement of different ER Ca^2+^ channels, the Ca^2+^ microdomains induced by adhesion of T cells before TCR/CD3 stimulation (ADCM) or by early NAADP signaling (TDCM) display similar characteristics in terms of amplitude and spatial extent because of the change in STIM isoform involved. Moreover, the model predicts that the flux at the mouth of the RYR1 or IP_3_R, which determines the extent of local Ca^2+^ depletion, rapidly becomes similar despite the largely different conductance of these channels, due to the slow Ca^2+^ diffusion in the ER.

### Towards a comprehensive model of Ca^2+^ signaling in the early phases of T cell stimulation

Based on these findings, T cell transition from quiescent to early activation can be described as schematized in **Figure 3C**. The resting state of a T cell (**Figure 3C**, left panel) is defined as an absence of any stimulatory contacts whatsoever, e.g. no contacts to cells, adhesive surfaces, or soluble activators; in this period, e.g. a T cell floating in gentle bloodstream, only basal (stochastic) openings of Orai1 channels are observed (**Figure 3C**, left panel). Upon weak (and likely transient) adhesion of the T cell, e.g. to proteins of the extracellular matrix during crossing of the basement membrane on the way to inflamed tissue areas, integrins are activated and cause stimulation of IP_3_R. STIM2 recruits STIM1 close to the junction under such low stimulation conditions (ADCM) (**Figure 3C**, middle panel). Following TCR/CD3 stimulation (TDCM), NAADP signaling activates RYR1 and newly formed STIM1/2 heterotetramers need greater local luminal depletion to trigger a quantitatively similar Ca^2+^ influx through Orai1 Ca^2+^ channels (**Figure 3C**, right panel). To best describe T cell transition and Ca^2+^ microdomains evolution in the ER-PM junction, a computational model should include both the stochastic character of channels opening and closing, and the description of Ca^2+^ diffusion and buffering. This would require to further include a molecular description of STIM/Orai1 interactions considering the formation of molecular complexes of different sizes and compositions (85–87). Moreover, the opening of IP_3_Rs or RYR1s in the cluster located near the junction should be described stochastically, as performed to describe Ca^2+^ puffs (88–90) or sparks (91). Mitochondria and PMCA’s should also be included, as in (57) and (60) The main challenges of such a model involve the formulation of the temporal shift from STIM1 to STIM2 pre-formed complexes with Orai1, and the interaction of ER Ca^2+^ channels IP_3_R and RYR1 when active simultaneously during the transition time. Importantly, current T cell Ca^2+^ imaging techniques (27, 43, 48) are not able to resolve single junction contributions to the formation of Ca^2+^ microdomains. The observed Ca^2+^ increases at the PM upon adhesion or TCR/CD3 stimulation may in fact result from the input of several junctions depending on how close they are positioned to each other. Mathematical modeling may cast some light into the possible conformations of the different channels participating to the formation of Ca^2+^ microdomains in a cluster of ER-PM junctions. This would also require a dynamical description of Ca^2+^ buffering that affects inter-cluster communication. Finally, continuous modelling of the evolution of a Ca^2+^ increase in the form of a microdomain to global Ca^2+^ signaling will require a more complex multiscale whole-cell model. Such a study will build on the existing whole cell model taking into account specific spatial geometries for mitochondria and the ER (60). Simulated geometries will also include well-defined numbers and locations of the different types of Ca^2+^ channels and pumps, as they have been inferred from the small-scale modeling of the ER-PM junctions. Alternatively, it might be as valid to develop a compartmental model where the microdomain is modelled as a separate compartment in which the concentrations change must faster than in the rest of the cytoplasm (92, 93). The global model should not only be hybrid (i.e. stochastic and deterministic), but its numerical simulation should be adaptive in time since it must consider different time and spatial scales (82). It needs to be able to resolve both the initial single-channel Ca^2+^ gating and the later shift to a modeling of the global Ca^2+^ increase due to clusters of Ca^2+^ channels. Despite all these modelling challenges, such global simulations are expected to enhance our understanding of the localized regulation of Ca^2+^ dynamics, and hence of Ca^2+^ signaling in which small local effects can be either damped or amplified at the cellular level.

## Concluding remarks

Computational modeling is increasingly used in cell biology to optimize the information that can be gained from the ever-improving techniques in imaging microscopy. In the field of ER-PM Ca^2+^ microdomains, this synergy has allowed to predict the number and spatial arrangement of Orai channels in the junction (18, 61), and to highlight the relatively minor role played by ER refilling by SERCA pumps in controlling the amplitude of the Ca^2+^ increase and the spatial spread of Ca^2+^ microdomains (62). When considering the ER Ca^2+^ channels surrounding the junction, a comparison between simulation results and high-resolution Ca^2+^ imaging uncovered the main role played by ER local depletion induced by weak IP_3_ or NAADP signaling in triggering the formation of adhesion-dependent and TCR/CD3-dependent Ca^2+^ microdomains in T cells (63, 64). In the future, it will be necessary to resort to observations-based multiscale modeling to dissect the relationship between local and global Ca^2+^ signaling, which could emphasize the key role played by the PMCA’s and the mitochondria in the T cell Ca^2+^ dynamics beyond the first seconds after stimulation (60). Despite the corresponding computational challenge, this effort is necessary to reveal how the versatile Ca^2+^ signaling pathway fine-tunes T cell immune responses.

## Author contributions

All authors listed have made a substantial, direct, and intellectual contribution to the work and approved it for publication.
